# Ipsilateral Oropharyngeal and Cervical Lymph Node Tuberculosis Simulating Oropharyngeal Malignancy with Regional Lymph Node Metastasis: A Case Report

**DOI:** 10.22038/ijorl.2025.85739.3877

**Published:** 2025

**Authors:** V Sha Kri Eh Dam, Nusaibah Azman

**Affiliations:** 1 *Department of Otorhinolaryngology-Head & Neck Surgery, Hospital Lahad Datu, Peti Surat 60065, 91110 Lahad Datu, Sabah, Malaysia.*; 2 *Department of Pathology, Hospital Queen Elizabeth 1, Karung Berkunci No. 2029, 88586 Kota Kinabalu, Sabah, Malaysia.*

**Keywords:** Tuberculosis, Oropharynx, Neoplasm

## Abstract

**Introduction:**

Tuberculosis (TB) is an important contagious disease and a major public health problem globally. It may manifest as pulmonary TB or primary or secondary extrapulmonary TB. Primary oropharyngeal TB is very rare and may mimic presentation of oropharyngeal malignancy especially in the negative initial TB workup.

**Case Report:**

We would like to highlight a case of an elderly man presented with ipsilateral oropharyngeal mass and cervical lymph node (LN) enlargement, mimicking oropharyngeal malignancy with regional LN metastasis.

**Conclusion:**

History of TB contact, poor oral hygiene, and poor immunity should alert the possibility of oropharyngeal TB. Involvement of ipsilateral oropharyngeal structure and cervical LN may simulate presentation of oropharyngeal malignancy with regional LN metastasis. Tissue biopsy for histopathological examination and appropriate staining is considered gold standard for diagnosis of TB and excluding malignancy. It is an important communicable disease, thus notification and referral to infectious disease team should be done without delay.

## Introduction

Tuberculosis (TB) infection is a major public health problem worldwide, especially in developing countries. In 2023, 8.2 million people were diagnosed with TB globally, and of these, 84% were pulmonary TB (PTB), and 16% were extrapulmonary TB (EPTB) (1). Lymph nodes (LN), especially those at the cervical region and pleura, are the most common sites for EPTB (2,3). By contrast, oral cavity and oropharyngeal involvements are rare and account for only 0.05%–5% of total TB cases (4,5). Tongue is the most common site for the oral cavity subsite (6), while tonsils are the most frequent site for the oropharyngeal subsite (3,5). The involvement of the ipsilateral oropharyngeal structure and cervical LN may simulate the presentation of oropharyngeal malignancy with regional LN metastasis. Although rare, concomitant TB and malignancy may occur and need to be confirmed or ruled out by histopathological examination (HPE) of the specimen. A systemic review of oral cavity TB showed that 3% of patients had concomitant carcinoma in the same lesion site (6).

## Case Report

**Fig 1:**
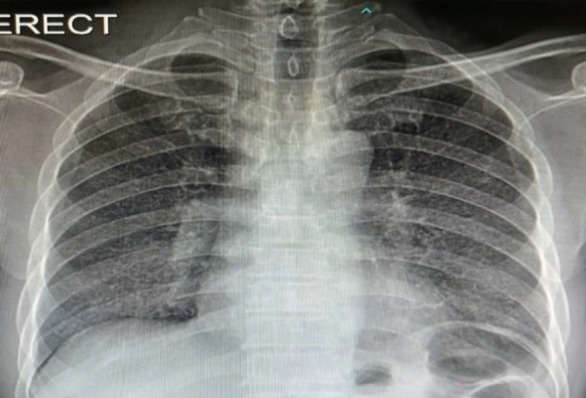
Normal chest x-ray without feature of tuberculosis

A 66-year-old male with underlying diabetic mellitus presented with right neck swelling for a duration of one month. The swelling was painless but progressively increased in size. It was associated with loss of appetite and weight, on and off fever and night sweats. There were no local obstructive symptoms, such as dysphagia or shortness of breath, and no changes in voice. The patient denied having a chronic cough or being in contact with TB patients. He was an active and chronic smoker but did not consume alcohol or engage in high-risk behaviour. There was no family history of malignancy. Screening for TB, including sputum for acid-fast bacilli, tuberculin skin test and chest X-ray (Figure 1), was performed at primary care before he was referred to us and was not suggestive of TB.

**Fig 2:**
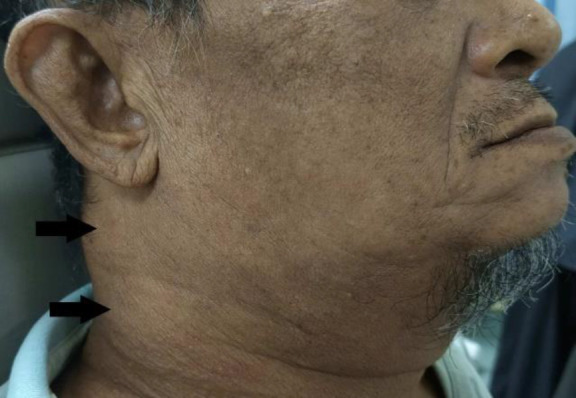
Multiple lymph nodes enlargement at the right side of the neck, at the level II, III and V.

**Fig 3:**
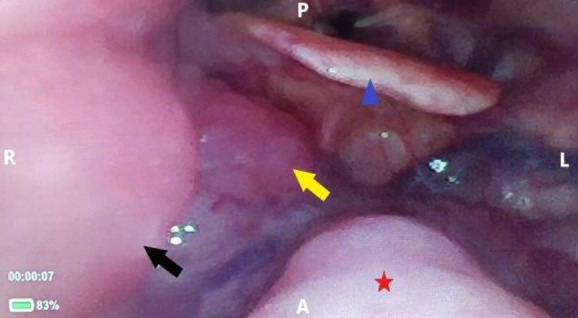
Flexible indirect laryngoscope shows unilateral right tonsil enlargement (black arrow) and a smooth surface mass at the right base of tongue (yellow arrow). Blue arrowhead – epiglottis; Red star – uvula; A – anterior; P – posterior; R – right; L- left.

On examination, there were multiple lymph nodes palpable on the right side of the neck at levels II, III and V (Figure 2). They were firm in consistency and mobile, with some of them being matted and non-tender. There was no palpable lymph node on the contralateral side. Oral examination revealed unilateral right tonsil enlargement, which appeared to have a smooth surface without ulceration or a fungating mass. The patient was partially edentulous and oral hygiene was poor. Flexible indirect laryngoscope showed unilateral right tonsil enlargement and a smooth surface mass at the right base of the tongue (Figure 3). Tongue mobility was normal, and other head and neck examinations were unremarkable.

**Fig 4:**
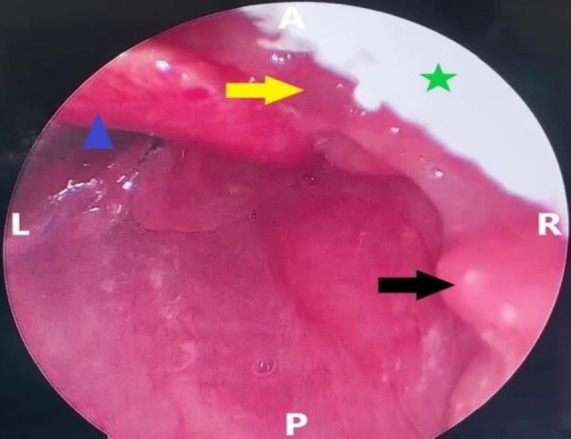
Direct laryngoscopy shows the mass at the right base of tongue (yellow arrow) is continuous with the right tonsil (black arrow). Blue arrowhead – epiglottis; Green star – anterior tongue; A – anterior; P – posterior; R – right; L- left.

Subsequently, the patient underwent direct laryngoscopy under general anaesthesia for a more detailed examination and biopsy. Direct laryngoscopy revealed that the mass at the right base of the tongue was continuous with the right tonsil (Figure 4). 

**Fig 5:**
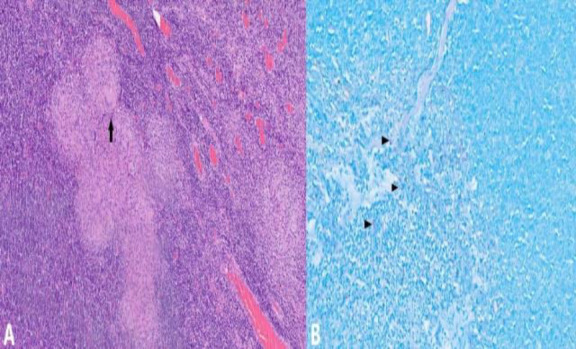
Multiple granulomas coalesce together in the background of reactive lymphoid cells and multinucleated giant cells of Langhans-type (arrow) (Fig A, H&E x200). Ziehl-Neelsen stain shows multiple acid-fast bacilli which are bright red coloured due to carbol-fuschin dye (arrowhead), while the background is methylene blue counterstain (Fig B, x200).

The mass had a smooth surface, was firm in consistency and was confined to the right oropharynx. The contralateral side, larynx, hypopharynx and anterior 2/3 of the tongue were not involved. A right tonsillectomy and biopsy of the right base of the tongue mass were performed. A computed tomography scan was not performed prior to the diagnostic tonsillectomy and biopsy because of limited resources and difficulty in obtaining an early CT scan at our centre. However, we planned to do so later if the HPE confirmed malignancy for staging purposes.The HPE of the right tonsil showed lymphoid tissue lined by stratified squamous epithelium with multiple foci of granulomatous formation, few foci of central suppurative material and necrosis, and more than 30 bacilli were seen using Ziehl–Neelsen stain (Figure 5). The HPE of the right base of the tongue mass showed reactive lymphoid tissue lined by squamous epithelium without granuloma. No atypical lymphocytes or malignancy was seen in either sample. Thus, the HPE findings were suggestive of TB of the right tonsil and reactive lymphoid hyperplasia of the right base of the tongue. We performed a through-cut biopsy of the right cervical lymph node to exclude the possibility of concomitant malignancy. The HPE was also consistent with TB, with the presence of necrotising granulomatous inflammation and acid-fast bacilli. The patient was referred to an infectious disease team for EPTB management.

## Discussion

TB is a chronic infectious granulomatous disease caused by acid-fast bacillus organisms, primarily *Mycobacterium tuberculosis*. The main route of transmission is respiration, and the lung is the main affected site. In the majority of cases, the infection remains confined to the lungs as it is controlled by the host immunity system, but it may also spread to other organ systems through the lymphatic system, blood stream or self-inoculation of infected sputum (4). TB can infect any part of the body, but the oral cavity and oropharyngeal involvement are considered rare sites. Most of the cases are in the form of secondary infections, mainly from the lung. 

The good protective mechanism of the upper respiratory tract prevents TB infection in these regions (3,7,8). The oral cavity and oropharynx have a thick epithelium lining that provides an important anatomical barrier from tuberculous bacilli inoculation. Furthermore, saprophytes in the saliva have a phagocytic property towards the bacilli. Saliva also has a cleansing action and contains enzymes with microbicidal action and immunoglobulin A. These mechanisms work synergistically to inhibit the growth and multiplication of the bacilli in these regions. 

The risk factors for oropharyngeal TB are poor immunity, poor oral hygiene, periodontitis, recent tooth extraction, leucoplakia and any breach in the oropharyngeal mucosa (8,9). Historically, *Mycobacterium bovis *was the main organism for oropharyngeal TB in the pre-pasteurisation era because of the ingestion of unpasteurised cow milk (10). However, this occurrence was markedly reduced after the introduction of antitubercular drugs and pasteurisation. Underlying diabetes mellitus and poor oral hygiene could be risk factors in this presenting case. The presentations of oropharyngeal TB vary and may simulate oropharyngeal malignancy, as seen in the presenting case. The most common symptoms of oral cavity and oropharyngeal TB are sore throat and odynophagia, while ulcerative lesions are the most frequent signs encountered (5,6). 

Fever, malaise, reduced appetite and weight loss are commonly associated constitutional symptoms. Collectively, these signs and symptoms are similar to oropharyngeal malignancy, particularly squamous cell carcinoma. Other less common oropharyngeal malignancies, such as minor salivary gland carcinoma and lymphoma, usually present with a painless submucosal mass. LN metastasis is early due to the rich lymphatic drainage in these regions. Because of the shared presentation, the unilaterality of the lesion, the negative TB contact and workup and the presence of risk factors (e.g. active chronic smoking), our initial diagnosis was more favoured towards malignancy. A tissue biopsy for HPE and appropriate staining is considered the gold standard for the diagnosis of TB, with the exclusion of malignancy. 

The typical pathological features of TB are epithelioid cells rimmed by lymphocytes, caseation necrosis and Langhans-type multinucleated giant cells, supported by the presence of acid-fast bacilli on the Ziehl–Neelsen stain (3–5). However, bacilli may not be detected, especially in small specimens with a reported detection rate of 27%–60% (4). The absence of atypical or malignant cells helps exclude malignancy. It is also important to exclude associated PTB, as primary oropharyngeal TB is less common than secondary TB. 

TB is an important global communicable disease and should be disclosed as soon as possible. Cases should be referred to the infectious disease team without delay to begin the appropriate anti-tuberculosis therapy. 

## Conclusion

Primary oropharyngeal TB is very rare and may mimic presentation of oropharyngeal malignancy. High suspicious should be made in patient with history of TB contact, poor oral hygiene, and has poor immunity like diabetes mellitus, human immunodeficiency virus infection and chronic alcoholism. Tissue diagnosis by HPE and appropriate staining are gold standard for diagnosis and excluding malignancy. It is an important communicable disease, thus notification and referral to infectious disease team should be done without delay.
